# Supplementary material for: HD-SiO2/SiO2 Sol@PDMS Superhydrophobic Coating with Good Durability and Anti-Corrosion for Protection of Al Sheets
Source: Materials (Basel). 2023 May 5;16(9):3532. doi: 10.3390/ma16093532 (PMC10180274; doi:10.3390/ma16093532)
Supplement: Supplementary file 1 [file materials-16-03532-s001.zip › materials-2329461-supplementary.docx]

Supporting Information for

HD-SiO_2_/SiO_2_ Sol@PDMS Superhydrophobic Coating with Good Durability and Anti-Corrosion for Protection of Al Sheets

**Ruohan Xia, Bing Zhang, Kousuo Dong, Yao Yan and Zisheng Guan ***

College of Materials Science & Engineering, Nanjing Tech University, 30 South PuZhu Road, Nanjing, Jiangsu, 211816, People’s Republic of China

* Correspondence: zsguan@njtech.edu.cn; Tel.: +86-138-1393-2186

**Table S1.** Content of each component in different SiO_2_ content experiments.

| **SiO_2_ wt.%** | **SiO_2_ content (g)** | **PDMS content (g)** | **TEOS**  **content (mL)** | **HDTMS content (mL)** | **WCA** |
| --- | --- | --- | --- | --- | --- |
| 0.0 wt.% | 0.0 g | 2 g | 1.5 mL | 0.4 mL | 82˚ |
| 0.2 wt.% | 0.1 g | 2 g | 1.5 mL | 0.4 mL | 115˚ |
| 0.4 wt.% | 0.2 g | 2 g | 1.5 mL | 0.4 mL | 142˚ |
| 0.6 wt.% | 0.3 g | 2 g | 1.5 mL | 0.4 mL | 150˚ |
| 0.8 wt.% | 0.4 g | 2 g | 1.5 mL | 0.4 mL | 158.5˚ |
| 1.0 wt.% | 0.5 g | 2 g | 1.5 mL | 0.4 mL | 158.5˚ |

**Table S2.** Content of each component in different TEOS content experiments.

| **TEOS wt.%** | **SiO_2_ content (g)** | **PDMS content (g)** | **TEOS**  **content (mL)** | **HDTMS content (mL)** | **WCA** |
| --- | --- | --- | --- | --- | --- |
| 1 wt.% | 0.4 g | 2 g | 0.5 mL | 0.4 mL | 151˚ |
| 2 wt.% | 0.4 g | 2 g | 1.5 mL | 0.4 mL | 158.5˚ |
| 3 wt.% | 0.4 g | 2 g | 3.0 mL | 0.4 mL | 156˚ |
| 4 wt.% | 0.4 g | 2 g | 4.5 mL | 0.4 mL | 142˚ |
| 5 wt.% | 0.4 g | 2 g | 5.5 mL | 0.4 mL | 140˚ |

**Table S3.** Content of each component in different PDMS content experiments.

| **PDMS wt.%** | **SiO_2_ content (g)** | **PDMS content (g)** | **TEOS**  **content (g)** | **HDTMS content (g)** | **WCA** |
| --- | --- | --- | --- | --- | --- |
| 1 wt.% | 0.4 g | 1.0 g | 1.5 mL | 0.4 mL | 158.5˚ |
| 2 wt.% | 0.4 g | 2.0 g | 1.5 mL | 0.4 mL | 158.5˚ |
| 5 wt.% | 0.4 g | 5.0 g | 1.5 mL | 0.4 mL | 82˚ |

**Figure S1.** FT-IR spectra of the PDMS and TEOS.

As shown in Figure S1, a strong absorption peak appears at 1108 cm^-1^ in the infrared spectrum of PDMS, which represents the stretching vibration of the Si-O bond; the characteristic peak at 810 cm^-1^ is the stretching vibration of the C-Si bond. In the spectrum of TEOS, the strong absorption peak appearing at 958 cm^-1^ is the stretching vibration of the Si-C bond; the absorption peak at 1011 cm^-1^ represents the stretching vibration of the Si-O bond.
